# Paralysis of the Portio Dura in an Infant

**Published:** 1844-04-20

**Authors:** 


### PARALYSIS OF THE PORTIO DURA IN AN INFANT.

At a meeting of the Westminster Medical Society, Jan. 20th, 1844, Dr. Chowne detailed a case of paralysis of the portio dura, affecting the face in the usual way, under which a child had laboured as early as the tenth month of his age.

James Loft, of fair complexion, blue eyes, and light hair, was supposed by his mother to be more than usually backward in his attempts to speak, and under these circumstances became Dr. Chowne's patient. The child at the time was twenty-two months old, and had eighteen teeth. The face was drawn a little to the left side; this was visible although not in a strong degree, when the child was still, but when he smiled it became very visible and while crying it amounted to a strong distortion. When the child was played with and laughed, the face was also distorted; the angle of the mouth being drawn to one side, the upper lip on the same side was drawn a great deal up, leaving the mouth very much open on the left side, while it was nearly closed on the right.

When the child was unexcited and the face still, the eyes appeared nearly alike as to degree of openness; there was, however, generally if not always, a greater accumulation of tears on the surface of the eye, and resting on the under lid of the right eye than on that of the left. When the finger was put near the eyelashes of the right eye the palpebræ closed partly but not quite, while those of the left closed perfectly at the same time without any other excitement than that applied to the right. When, however, the finger touched the eyelashes the palpebræ of the right eye also closed completely. During sleep the left eye was closed, the right partly open.

During both laughing and crying the right side of the face was particularly still, dull, and inexpressive while the left was in full activity and animation. The child was intelligent and playful, and when played with and tickled laughed heartily; at these times the contrast between the two sides of the face was remarkably striking and characteristic; one side was full of expression and mirth, while the other presented a sombre, dull, inanimate stillness, wholly devoid of participation in the mirth enjoyed by the child, and depicted in the merry side of his countenance. This appearance, even in so young a child, was very grotesque.

The mother had not noticed anything particular in the child until about ten months prior to Dr. Chowne's seeing it; that is to say, not until the child, was ten months old. At that time the state of the face was pointed out to her by her friends; not by friends, however, who had been in the habit of seeing the child. Those friends and acquaintances who, like the mother, had seen the child frequently, or almost constantly had not noticed the peculiarity; hence it is not certain at what time the malady first existed.

The mother was not aware that the child had received any injury, neither had it had any illness which had even attracted her attention to the head, or ear, or face, particularly.

At the time the child was seen by Dr. Chowne alterative aperient medicines were prescribed, and all appearance of indisposition passed away, so far as the health was concerned, but the paralysis remained unaltered.

Dr. Chowne observed that paralysis of the portio dura was a very unusual affection in so young a subject. He was quite unable to decide upon the cause of the affection in this case. In answer to a question, he said the labour in the case of this child was not a difficult one.

A discussion of some length followed between various members of the society, chiefly with reference to the pathology of cases of paralysis of the portio dura, in which various opinions were expressed, but none of them require to be separately detailed. It may be mentioned, however, that Dr. Reid had seen two cases of partial paralysis of this nerve consequent upon the application of the forceps to the child's head during labour.—*Ibid.*

